# Impact of cryotherapy on incidence and severity of oral mucositis in hematopoietic stem cell transplantation: retrospective observational study

**DOI:** 10.1038/s41598-025-20927-5

**Published:** 2025-10-23

**Authors:** Oliwia Piotrowska, Alina Zuchmańska, Piotr Kulig, Tomasz Michowski, Jacek Szulc, Patryk Skórka, Dominika Dorsz, Bogusław Machaliński, Sławomir Milczarek

**Affiliations:** 1 Department of Physiopathology, Hematology and Transplantology, Pomeranian Medical University in Szczecin, 71-252 Szczecin, Poland; 2 University Pharmaceutical Manufactory Pomeranian Medical University in Szczecin, 71-251 Szczecin, Poland

**Keywords:** Oral mucositis cryotherapy, Hematopoietic stem cell transplantation, Chemotherapy, Radiotherapy, Side effects, Haematopoietic stem cells, Chemotherapy

## Abstract

Oral mucositis (OM) is a common and significant complication of chemotherapy and radiotherapy, particularly in patients undergoing hematopoietic stem cell transplantation (HSCT). This retrospective observational study assessed the effectiveness of oral cryotherapy in reducing the incidence and severity of OM in 258 HSCT recipients treated at a single transplant center in Poland between 2018 and 2024. Patients were divided into two groups: 199 received cryotherapy as part of their conditioning regimen, while 59 did not. Cryotherapy was administered using ice chips from 10 min before chemotherapy infusion to two hours afterward. OM occurred in 48% of the cryotherapy group versus 68% in the control group, representing a statistically significant 20% relative risk reduction (*p* = 0.008). Furthermore, the incidence of severe OM (grades 3–4) was significantly lower in the cryotherapy group (*p* = 0.006). No significant differences were observed in platelet engraftment or hospitalization duration. Although neutrophil engraftment was slightly delayed in the cryotherapy group, this finding lacked clinical relevance. Overall, the results support cryotherapy as a safe, cost-effective, and easily implementable strategy for OM prevention in the HSCT setting. Further prospective studies are warranted to validate these findings and optimize treatment protocols.

## Introduction

Hematopoietic stem cell transplantation (HSCT) represents a complex therapeutic strategy for the management of hematological malignancies^1^ and selected non-hematological disorders^2,3^. In recent years, there has been a consistent increase in the total number of HSCTs performed, accompanied by the emergence of a broader spectrum of conditioning regimens^4^. The conditioning phase, which is essential before the HSCT, may involve the utilization of chemotherapy and radiation therapy, including total body irradiation (TBI)^5^. The short- and long-term adverse effects associated with this treatment are significant and include myelotoxicity, mucositis, nausea, vomiting, diarrhea, cutaneous rashes, alopecia, and peripheral neuropathies^1^.

Oral mucositis (OM), an ulcerative and inflammatory disease of the oral mucosa, is recognized as one of the most prevalent complications in patients undergoing chemotherapy and/or radiotherapy. OM is associated with direct stoma toxicity and usually develops within 3 to 7 days after chemotherapy and resolves within approximately 2 to 3 weeks, in parallel with neutrophil recovery. The incidence of OM is associated with heightened morbidity and can adversely influence treatment adherence and overall patient outcomes. The presentation of OM varies according to the severity grade. Clinical manifestations may include alterations in taste perception, restricted range of motion in the jaw, difficulties in swallowing and speaking, and disturbances in sleep patterns.

The incidence of mucositis in patients undergoing hematopoietic stem cell transplantation (HSCT) is closely linked to the exacerbation of infectious and nutritional complications. By compromising the integrity of the mucosal barrier, mucositis is a potential conduit for the onset of bloodstream infections. These lesions can be portals for systemic infections, including bacteremia^6,7^, pose significant risks to patient health and extend the duration of hospitalization substantially. The management of these complications often necessitates intravenous antibiotic therapy and, in some cases, the administration of vasoconstrictor agents, thereby escalating the overall costs associated with patient care and hospitalization^8–11^.

Mucositis is frequently correlated with heightened levels of pain in patients, which subsequently diminishes their quality of life and necessitates the administration of supplementary analgesics^12^. The implementation of cryotherapy during the conditioning phase of hematopoietic stem cell transplantation has the potential to significantly reduce the requirement for pain management, particularly the utilization of opioids. Opioids themselves are associated with a range of adverse effects, including constipation, nausea, and vomiting. Furthermore, by mitigating both the incidence and severity of mucositis, we can enhance patient comfort and overall quality of life (QoL)^11,13^.

The pain itself may lead to the development of anorexia and then malnutrition, which may prolong the patient’s stay in the hospital. All this together leads to the subsequent need to implement TPN (Total Parenteral Nutrition).

The prevalence and severity of OM could vary depending on the type of transplant and conditioning regimen. The incidence of severe oral mucositis (SOM) is greater in patients undergoing alloSCT compared to autoSCT recipients due to prolonged neutropenia in the alloSCT group. SOM frequency reached 98% in the group receiving a conditioning regimen consisting of total body irradiation^14^. In a single-center retrospective study by Nakagaki et al., SOM (grade 3–4) was common in myeloablative total body irradiation (TBI) based regimens cyclophosphamide/TBI (71%) and fludarabine/TBI with PTCy (46%). In contrast, SOM occurred less frequently in reduced-intensity haploidentical regimen melphalan/fludarabine/TBI with PTCy (19%)^15^. Multicentre and retrospective study by L. Vagliano and colleagues demonstrated that 71% of patients who underwent HSCT were diagnosed with OM, and 21.6% developed SOM^16^.

Moreover the duration and severity of mucositis are associated with the intensity of conditioning - they are both diminished in RIC setting^14,12^. Increased rates of OM were found in 86,5% HSCT patients who received myeloablative regimens versus 73.2% of patients who received reduced-intensity regimens. HSCT conditioning regimens, including those that utilize melphalan, busulfan, or radiotherapy, are significant contributors to the pathogenesis of oral mucositis^17^.

Various interventions have been tested and implemented for the prevention and treatment of oral mucositis (OM)^6,7,18,19^. The Mucositis Study Group of the Multinational Association for Supportive Care in Cancer and the International Society of Oral Oncology (MASCC/ISOO) has provided clinical guidelines for managing mucositis^20^. Oral cryotherapy is commonly used to prevent OM, with minimal risk of side effects. This therapy involves placing ice in the form of ice cubes in the mouth before or during chemotherapy treatment^21,22^. The cooling effect causes local hypothermia, leading to vasoconstriction of the oral mucosal blood vessels, which decreases blood flow to the tissues. As a result, the concentration of cytotoxic agents in the oral mucosa is reduced. Additionally, oral cryotherapy may lower the metabolic rate in the oral epithelium, helping to decrease inflammation.

Following the increasing incidence of mucositis, new clinical practice guidelines for managing mucositis secondary to cancer therapy were published by MASCC/ISOO in 2020. The safety and effectiveness of recombinant human keratinocyte growth factor-1 (KGF-1/palifermin) in OM prevention have been established. Palifermin has proven effectiveness in significantly reducing the incidence and duration of grade 3–4 OM after myeloablative therapy. Furthermore, apart from palifermin, there are two human fibroblast growth factors (velafermin and repifermin), which may open the door to a focused strategy for mucositis prevention^23,24^.

Limited data on the successful OM treatment in patients receiving HSCT are available. Morphine is indicated for pain treatment due to oral mucositis in patients undergoing HSCT. Borowski et al. state in their article that a 2% morphine oral mouthwash may be used to treat mucosal pain accompanying OM^25^.

## Materials and methods

### Study design

#### Patients

From 23.07.2018 to 01.02.2024, a retrospective observational study was conducted at the University Clinical Hospital No. 1 of the Pomeranian Medical University in Szczecin (Poland). We investigated 258 consecutive autologous and allogeneic peripheral blood stem cell transplantation (PBSCT) procedures. All patients suffered from hematological malignancies or solid tumors. Detailed patients and cryotherapy characteristics are presented in Table 1 in the Results section.

**Table 1:** Patient and mucositis characteristics.

		Cryotherapy (+) *n* = 199		Cryotherapy (−) *n* = 59	
		Mucositis (+) *n* = 95 (48%)	Mucositis (−) *n* = 104 (52%)	Mucositis (+) *n* = 40 (68%)	Mucositis (−) *n* = 19 (32%)
Grade—CTCAE V	G1/G2	89	–	34	–
	G3/G4	6	–	6	–
Age (years)	Mean (x̄)	52,27 (σ = 12,87)	56,90 (σ = 11,93)	63,33 (σ = 8,78)	61,58 (σ = 8,75)
	Median	56	61	65	63
Sex	Male/Female	113 (57%)/86 (43%)		30 (51%)/29(49%)	
Hospital stay duration	Mean (x̄)	27,11 (σ = 13,13)	24,27 (σ = 6,80)	25,08 (σ = 8,00)	23,32 (σ = 6,25)
	Median	24	22	22	21
	Plasma Cell dyscrasias (PCM + PCL)	105		50	
	Acute leukemias (AML+ALL+ blast crisis)	22		–	
	MDS/MPN (CMML+ MDS/AML+AML)	4		–	
	NHL (LL+DLBCL+FL+PCNSL+MCL+PTCL+HGBCL+BL+WM)	39		8	
	HL	14		1	
	SGF	1		–	
	GCT	13		–	
	BPDCN	1		–	
Auto/AlloSCT	Auto	76	88	40	19
	Allo	19	16	–	–
	MAC allo	13	3	–	–
	Non-MAC allo	6	13	–	–
TPN	Yes	32	26	11	7
	No	63	78	29	12

G1-4– mucositis stage according to CTCAE, PCM-plasma cell myeloma, AML-acute myeloid leukemia, ALL-acute lymphoblastic leukemia, PCL-plasma cell leukemia, CMML-chronic myelomonocytic leukemia, MPN- myeloprofiferative neopalsm, MDS–myelodysplastic syndrome, LL-lymphoblastic lymphoma, DLBCL-diffuse large B-cell lymphoma, WM-Waldenström macroglobulinemia, FL-follicular lymphoma, BL-Burkitt lymphoma, NHL- Non- Hodgkin lymphoma, HL-Hodgkin lymphoma, PCNSL-primary central nervous system lymphoma, MCL–mantle cell lymphoma, PTCL- peripheral T-cell lymphoma, SGF- secondary graft failure GCT—germ cell tumor, BPDCN-blastic plasmacytoid dendritic cell neoplasm, HGBCL-high-grade B-cell Lymphoma, alloSCT-allogeneic stem cell transplantation, autoSCT-autologous stem cell transplantation, MAC-myeloablative conditioning regimen, Non-MAC- non myeloablative conditioning regimen, TPN- total parental nutrition.

The participants in the study were categorized into two distinct groups: the study group comprised 199 patients who underwent cryotherapy as part of their conditioning regimen, whereas the control group included 59 patients who did not receive cryotherapy. Since 2020, there has been a significant increase in scientific literature highlighting the effectiveness of cryotherapy in the prevention of mucositis. In response to this body of evidence, we have incorporated cryotherapy into our standard operating procedures, implementing it as a crucial component of conditioning therapy for all patients scheduled to undergo hematopoietic stem cell transplantation (HSCT). Consequently, our control group comprises patients who underwent HSCT prior to September 2020, during which the treatment protocol did not include cryotherapy as part of the conditioning regimen.

The study was approved by the Bioethics Committee of the Pomeranian Medical University in Szczecin. The study was retrospective in nature, so an opinion from the local bioethics committee is not necessary. For those interested, the bioethics committee issued a statement stating that the study did not require its consent, which we received. No organs were harvested from prisoners in the study. Both donors and recipients of hematopoietic stem cells signed informed consents before the procedures were performed. All the research involving our participants was performed in accordance with the Declaration of Helsinki.

#### Method of performing cryotherapy

The cryotherapy procedure is conducted as follows: prior to initiation, the physician informs the patient about the procedure and its clinical purpose. Cryotherapy is currently standard procedure for all patients during conditioning prior to HSCT. This procedure does not require a separate order from the treating physician. Ice chips are generated on the ward using an ice maker supplied with sterile water. Oral cryotherapy introduces various ice forms — such as chips, cubes, or ice-cold water. The nursing staff administer the ice chips in single-use cups, commencing 5 min before the start of chemotherapy infusion. Patients are instructed to perform oral cryotherapy by sucking the ice chips from 10 min prior to infusion, maintained throughout the duration of the infusion, and continued for up to 2 h following infusion completion. In cases where patients experience headache or discomfort, brief interruption of cryotherapy is permitted, with resumption advised as soon as tolerated. CTCAE mucositis assessment was performed daily during the patient’s hospitalization by the attending physicians after a prior physical examination, which was then included in the medical observations and epicrisis in the section on therapy complications. Apart from cryotherapy, the study group and the control group had oral care protocols implemented. Basic oral care protocols generally include brushing teeth, flossing, and at least one mouth rinse to uphold oral hygiene. Each patient with MAC conditioning prior to the alloHSCT procedure additionally received methotrexate as prophylaxis against GvHD, which could also cause damage to the oral mucosa.

#### Statistical analysis

Continuous variables are expressed as mean (median); interquartile range (IQR). The Shapiro-Wilk test was implemented to assess the distribution of continuous variables. As constant variables were not normally distributed, the Mann-Whitney U test was implemented to compare the differences between the groups. Fisher exact test was used to assess differences in the categorical variables between the groups. When relevant post hoc pairwise comparison with Bonferroni correction for multiple testing was implemented. P value < 0.05 was considered statistically significant. All calculations were performed in RStudio.

#### Definitions

According to CTCAE, oral mucositis is defined as a disorder characterized by ulceration or inflammation of the oral mucosa. The severity of oral mucositis was defined as per the CTCAE oral toxicity scale, separating mucositis into 5 Grades. Grade 1 = Asymptomatic or mild symptoms; intervention not indicated, Grade 2 = oral ulcers, patients able to take solids; Grade 3 = Severe pain; interfering with oral intake, Grade 4 = Life-threatening consequences; urgent intervention indicated; Grade 5 = Death.

## Results

### Characteristics of the study group

The characteristics of the group are presented in Table 1.

### Cryotherapy reduces the incidence rate of oral mucositis

Cryotherapy has been demonstrated to significantly reduce the incidence of oral mucositis (OM) among recipients of stem cell transplants, regardless of the transplant modality employed (as shown in Table 1). Our investigation revealed that 48% of patients undergoing cryotherapy developed OM, in stark contrast to a 68% incidence observed in patients who did not receive this intervention. This finding corresponds to a relative risk reduction of 20% (RRR 20%, *p* = 0.008). Furthermore, cryotherapy has been shown to mitigate the risk of developing severe oral mucositis, specifically in grades 3–4 (*p* = 0.006).

Post-hoc analysis further elucidated statistically significant differences between individuals who did not develop OM and those who experienced OM graded as G1-2 and G3-4, with p-values of 0.044 and 0.029, respectively.

In our investigation, we did not observe statistically significant differences regarding the length of hospitalization (*p* = 0.37) or platelet engraftment (*p* = 0.537). It is noteworthy that neutrophil engraftment within the cryotherapy cohort was observed to occur slightly later compared to the non-cryotherapy cohort, with mean durations of 12.38 days and 11.63 days, respectively (*p* = 0.045) (Table 2).

**Table 2:** Analysis of thrombo- and myelopoiesis regeneration in patients after autosct and allosct transplants in the group after cryotherapy and without intervention.

		AutoSCT		AlloSCT	
		Cryotherapy (+)	Cryotherapy (−)	Cryotherapy (+)	Cryotherapy (−)
Myelopoiesis regeneration (days after transplant)	Mean (x̄)	11,46 (σ = 2,11)	11,63 (σ = 2,27)	16,69 (σ = 4,82)	–
	Median	11	11	16	–
Thrombopoiesis regeneration (days after transplant)	Mean (x̄)	14,98 (σ = 6,10)	15,25 (σ = 5,84)	17,42 (σ = 11,30)	–
	Median	14	13	13	–

alloSCT- allogeneic stem cell transplantation, autoSCT- autologous stem cell transplantation.

However, this delay lacks clinical significance, as it does not correlate with an extension of the hospitalization period. It should be emphasized that the cryotherapy group was more diverse as it included patients undergoing alloSCT and patients treated for lymphomas where both subgroups are characterized by a more extended period of neutropenia compared to autoHSCT in myeloma. The control group included only patients undergoing the autoHSCT procedure, usually after melphalan conditioning for multiple myeloma (Table 3).

**Table 3:** Analysis of the incidence rate of oral mucositis between the patients who received cryotherapy and those who did not.

	Cryotherapy (*n* = 199)	No cryotherapy (*n* = 59)	OR	RRR	*p*
Mucositis yes/no	95/104	40/19	0.4	20%	**0.008**
Length of stay mean (median); IQR	25.62 (23), 19–29	24.51 (22), 19–25			0.37
Platelet engraftment mean (median); IQR	15.37 (14), 12–16	15.25 (13), 12-16.5			0.537
Neutrophil engraftment mean (median); IQR	12.38 (11), 11–12	11.63 (11), 11–12			**0.045**
Mucositis stage					
G1-2	89	34			**0.0056**
G3-4	6	6			
No mucositis	104	19			
Mucositis stage; post-hoc comparison					
G1-2/no mucositis	89/104	34/19			**0.044**
G3-4/no mucositis	**6/104**	**6/19**			**0.029**

Continuous variables were analyzed with Mann-Whitney U test while categorical variables with Fisher exact test which were applicable was followed by post-hoc pairwise comparison with Bonferroni correction for multiple testing. *p* < 0.05 was considered statistically significant.

*OR* odds ratio, *RRR* relative risk reduction, *TPN* total parenteral nutrition, *IQR* interquartile range, *G1-4* mucositis stage according to CTCAE, *MAC* myeloablative conditioning regimen, *RIC* reduced intensity conditioning regimen.

### Oral mucositis occurs less frequently in allosct patients with reduced intensity conditioning regimen

Next, we wanted to analyze if OM mucositis occurred more frequently in any subgroup regardless of cryotherapy (Table 4). We found no statistically significant difference in age between MC (mucositis) and nMC (non-Mucositis) patients (*p* = 0.2) and in the frequency of HSCT types, both considering the entire cohort (*p* = 0.86) and the group of patients who received cryotherapy (*p* = 0.45). Among alloSCT patients (all alloSCT individuals received cryotherapy) OM occurred less frequently in the RIC (reduced intensity conditioning) subgroup compared to the MAC (Myeloablative Conditioning) conditioning group (RRR 61%, *p* = 0.006) (Table 4).

**Table 4:** Analysis of the incidence rate of oral mucositis.

	Mucositis	No mucositis	OR	RRR	*p*
Age mean(median), IQR	55.55 (59), 46–65	57.63 (61), 51–66			0.2
autoSCT/alloSCT	116/19	107/16	0.91	1%	0.86
autoSCT/alloSCT (cryotherapy group)	76/19	88/16	0.73	5%	0.45
MAC/RIC (alloSCT)	13/6	3/13	0.11	61%	**0.006**

Continuous variables were analyzed with Mann-Whitney U test while categorical variables with Fisher exact test. *p* < 0.05 was considered statistically significant.

*OR* odds ratio, *RRR* relative risk reduction, *IQR* interquartile range, *MAC* myeloablative conditioning regimen, *RIC* reduced intensity conditioning regimen.

Analysis of the frequency of conditioning regimens and the incidence and severity of mucositis in patients receiving different types of conditioning regimens is presented in Table 5.

**Table 5:** Analysis of the frequency of conditioning regimens and the incidence and severity of mucositis in patients receiving different types of conditioning regimens.

Conditioning regimen	Patients (*n*) / % of patients	Mucositis (*n* of patients)	Mucositis G1/G2 (*n* of patients)	Mucositis G3/G4 (*n* of patients)
Beeam	53 / 20,54%	29	24	5
Cryotherapy (+)	44	22	20	2
Cryotherapy (−)	9	7	4	3
BeTMI	3 / 1,16%	2	2	0
Cryotherapy (+)	3	2	2	0
Cryotherapy (−)	0	0	0	0
BuTT	1 / 0,39%	1	1	0
Cryotherapy (+)	1	1	1	0
Cryotherapy (−)	0	0	0	0
BuCyE	5 / 1,94%	4	4	0
Cryotherapy (+)	5	4	4	0
Cryotherapy (−)	0	0	0	0
BuMEL	1 / 0,39%	1	1	0
Cryotherapy (+)	1	1	1	0
Cryotherapy (−)	0	0	0	0
CE	13 / 5,04%	3	3	0
Cryotherapy (+)	13	3	3	0
Cryotherapy (−)	0	0	0	0
CyTBI	2 / 0,78%	2	0	2
Cryotherapy (+)	2	2	0	2
Cryotherapy (−)	0	0	0	0
FluTBI	5 / 1,94%	4	4	0
Cryotherapy (+)	5	4	4	0
Cryotherapy (−)	0	0	0	0
FluBu2	9 / 3,49%	1	1	0
Cryotherapy (+)	9	1	1	0
Cryotherapy (−)	0	0	0	0
FluBu3	3 / 1,16%	2	2	0
Cryotherapy (+)	3	2	2	0
Cryotherapy (−)	0	0	0	0
FluBu4	7 / 2,71%	5	5	0
Cryotherapy (+)	7	5	5	0
Cryotherapy (−)	0	0	0	0
FluCy	1 / 0,39%	0	0	0
Cryotherapy (+)	1	0	0	0
Cryotherapy (−)	0	0	0	0
FluMEL	3 / 1,16%	1	1	0
Cryotherapy (+)	3	1	1	0
Cryotherapy (−)	0	0	0	0
FluTreo	1 / 0,39%	1	1	0
Cryotherapy (+)	1	1	1	0
Cryotherapy (−)	0	0	0	0
HYPER-CVAD	1 / 0,39%	1	0	0
Cryotherapy (+)	1	1	0	0
Cryotherapy (−)	0	0	0	0
MEL140	38 / 14,73%	16	16	0
Cryotherapy (+)	22	6	6	0
Cryotherapy (−)	16	10	10	0
MEL 200	111 / 43,02%	62	57	5
Cryotherapy (+)	77	39	37	2
Cryotherapy (−)	34	23	20	3
TBF	1 / 0,39%	1	1	0
Cryotherapy (+)	1	1	1	0
Cryotherapy (−)	0	0	0	0

*BeEAM* carmustine, etoposide, cytarabine and melphalan, *BeTMI* bendamustine, total marrow irradiation, *BuTT* Busulfan, Thiotepa, *BuCyE* busulfan, cyclophosphamide, etoposide, *BuMEL* busulfan, melphalan, *CE* carboplatin, etoposide, *CyTBI* cyclophosphamide, total body irradiation, *FluTBI* fludarabine, total body irradiation, *FluBu2* fludarabine, busulfan (2 days), *FluBu3* fludarabine, busulfan (3 days), *FluBu4* fludarabine, busulfan (4 days), *FluMEL* fludarabine, melphalan, *FluTreo* fludarabine, treosulfan, *HYPER CVAD* cyclophosphamide, vincristine, doxorubicin, methotrexate, cytarabine, dexamethasone, *MEL140* melphalan 140 mg/m^2^, *MEL200* melphalan 200 mg/m^2^.

## Discussion

Research has demonstrated that cryotherapy effectively reduces both the incidence and severity of oral mucositis in patients undergoing high-dose chemotherapy during hematopoietic stem cell transplantation (HSCT)^8,26,17^. In our study, we also confirmed this relationship, noting a 20% relative reduction in the risk of mucositis among patients who used cryotherapy compared to those who did not (RRR 20%, *p* = 0.008). Furthermore, post hoc analysis revealed that cryotherapy group less frequently develop mucositis G1-2 and G3-4 compared to non cryotherapy patients, *p* = 0.044 and *p* = 0.029 respectively. Moreover, cryotherapy has been shown to mitigate the risk of developing severe oral mucositis, specifically in grades 3–4 (*p* = 0.006).

In our research, we found no differences in the incidence of mucositis between alloSCT and autoSCT (*p* = 0.86). Notwithstanding, among alloSCT patients (all alloSCT individuals received cryotherapy) OM occurred less frequently in RIC subgroup (RRR 61%, *p* = 0.006). The absence of statistically significant differences in the incidence of mucositis between the autoSCT and alloSCT groups may be explained by the use of both RIC and MAC regimens in the alloSCT group, in contrast to the exclusive use of MAC regimens in the autoSCT group.

The use of cryotherapy supports the patient’s physiological alimentation of food, which helps reduce the incidence of thrombosis or infections in central vein access lines, TPN complications. However, in our patients, we did not demonstrate a relationship between the occurrence of mucositis and an increased risk of using TPN, which may be caused by a small study sample (*p* = 0.87).

According to some publications, e.g. “*Current Trends in Management of Oral Mucositis in Cancer Treatment”* by Shankar A. and colleagues, mucositis leads to prolonged regeneration of hematopoiesis, including myelopoiesis and thrombopoiesis as well as prolonged hospitalization^6^. The findings of our study did not demonstrate a statistically significant relationship between the use of cryotherapy and the duration of stem cell engraftment or the length of hospital stay (p = 0.37). As mentioned earlier, this is most likely related to the characteristics of the study and control groups - the cryotherapy group included patients undergoing alloSCT and patients treated for lymphomas, where both subgroups are characterized by a longer period of neutropenia compared to classic autoSCT recipients and have longer hospitalization time. Further studies on the effectiveness of cryotherapy are required, comparing groups of patients homogeneous in terms of diagnosis and transplant type.

Presented study has certain limitations. The incidence, duration, and severity of mucositis may be affected in the MAC alloHSCT group by a short course of intravenous methotrexate which is routinely administered for graft-versus-host disease prophylaxis in this clinical context. Furthermore, the study was retrospective, which may have mitigated our ability to control unpredicted confounders. Another important limitation of this study is the heterogeneity of the patient group. Participants differed in terms of underlying diagnoses, disease status at the time of hematopoietic stem cell transplantation (HSCT), prior treatment exposure, including previous chemotherapy, the number of prior therapy lines, type of transplantation (autoSCT vs. alloSCT), and conditioning regimens. The control group consisted primarily of patients undergoing autoSCT for multiple myeloma or relapsed/refractory lymphomas, whereas patients which underwent alloSCT, who were part of the study group, experienced significantly longer durations of neutropenia and hospital stays. This variability may have introduced confounding factors that limit the generalizability and comparability of the results. Further studies conducted on homogeneous groups of patients and focusing on the use of cryotherapy in mucositis prevention in patients undergoing conditioning prior to SCT are needed to confirm our findings.

In conclusion, the application of cryotherapy during the conditioning phase for patients during the HSCT procedure represents a highly economical approach to the prevention of mucositis, which yields several advantages for patient care. Consequently, we advocate for the routine implementation of cryotherapy in all patients undergoing hematopoietic stem cell transplantation.

## Data Availability

The datasets generated during and/or analysed during the current study are available from the corresponding author on reasonable request.
